# JAK-STAT Pathway: A Novel Target to Tackle Viral Infections

**DOI:** 10.3390/v13122379

**Published:** 2021-11-27

**Authors:** Ifeanyi Jude Ezeonwumelu, Edurne Garcia-Vidal, Ester Ballana

**Affiliations:** AIDS Research Institute-IrsiCaixa and Health Research Institute Germans Trias i Pujol (IGTP), Hospital Germans Trias i Pujol, Universitat Autònoma de Barcelona, 08916 Badalona, Spain; iezeonwumelu@irsicaixa.es (I.J.E.); egvidal@irsicaixa.es (E.G.-V.)

**Keywords:** JAK-STAT, innate immunity, antiviral, inflammation, therapeutic strategies, COVID-19, treatment

## Abstract

Modulation of the antiviral innate immune response has been proposed as a putative cellular target for the development of novel pan-viral therapeutic strategies. The Janus kinase–signal transducer and activator of transcription (JAK-STAT) pathway is especially relevant due to its essential role in the regulation of local and systemic inflammation in response to viral infections, being, therefore, a putative therapeutic target. Here, we review the extraordinary diversity of strategies that viruses have evolved to interfere with JAK-STAT signaling, stressing the relevance of this pathway as a putative antiviral target. Moreover, due to the recent remarkable progress on the development of novel JAK inhibitors (JAKi), the current knowledge on its efficacy against distinct viral infections is also discussed. JAKi have a proven efficacy against a broad spectrum of disorders and exhibit safety profiles comparable to biologics, therefore representing good candidates for drug repurposing strategies, including viral infections.

## 1. Introduction

**Figure 1:**
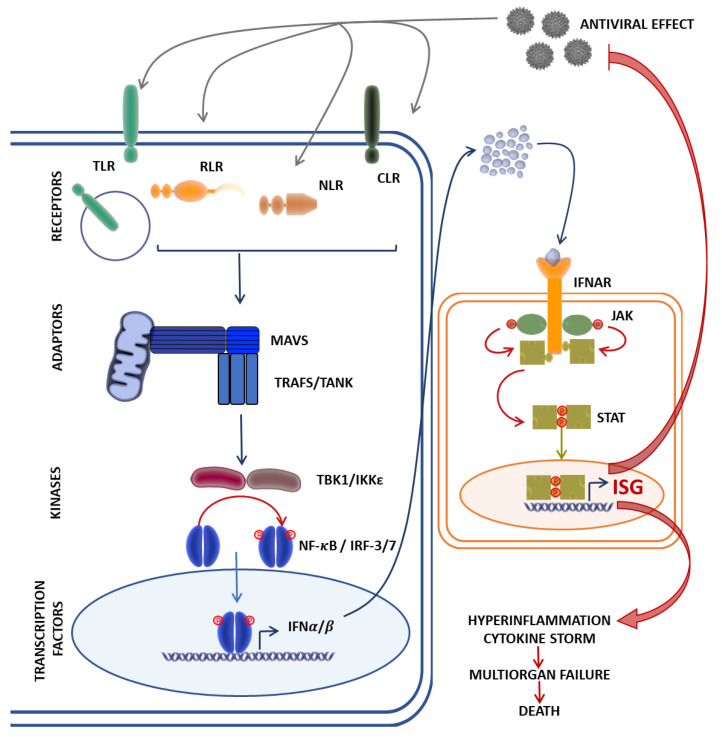
The Janus kinase–signal transducer and activator of transcription (JAK-STAT) signaling pathway mediates the innate immune response against viral infection. Cytosolic viral RNA recognition by pathogen recognition receptors such as the Toll-like receptors (TLR), RIG-I-like receptors (RLR), NOD-like receptors (NLR), and the C-type lectin receptors (CLR) induce interactions with the mitochondrial antiviral signaling protein (MAVS) and its co-adaptor molecules, TRAFs, which, acting through the TBK1/IKKε axis, activate nuclear factor-κB (NF-κB) and interferon regulatory factor (IRF-3/IRF-7) expression of type I interferons (IFNα/β). Type I IFN recognition and binding to IFNα receptors (IFNAR) activates JAKs, leading to the phosphorylation and translocation of STATs to the nucleus, resulting in the target gene expression of antiviral ISGs, and proinflammatory cytokines by transcriptional factors NF-κB and IRFs. A dysregulated immune response in rare cases might result in fatal outcomes due to hyperinflammation and the cytokine storm. ISG, interferon-stimulated gene; RIG-I, retinoic acid-inducible gene I; TRAF, tumor necrosis factor receptor-related factor; TBK1, TANK-binding kinase 1; IKKε, IκB kinase ε.

Innate immunity acts as our first line of defense for the detection and clearance of viral infections. Immediately after infection, all viruses trigger an antiviral response that relies on elements of innate immunity, such as physical barriers and the production of interferons (IFN) and groups of cytokines, a process orchestrated by innate immune cells—in particular, monocytes/macrophages, dendritic cells (DC), and natural killer (NK) cells [1,2]. Upon viral entry into target cells, pattern-recognition receptors (PRR) recognize the viral components and prompt IFN production. The secreted IFNs bind to their respective receptors and activate the Janus kinase–signal transducer and activator of transcription (JAK-STAT) pathway [3], resulting in the production of hundreds of downstream antiviral IFN-stimulated genes (ISGs) and the secretion of proinflammatory cytokines. This process establishes an antiviral state that inhibits viral replication, stimulates the adaptive immune response, and recruits other immune cells to the site of infection [4,5] (Figure 1).

Although optimal activation of the innate immunity in the course of viral infection is very important for viral clearance, an acute viral infection can also lead to disease progression through immune-mediated host tissue injury. Secreted proinflammatory cytokines can cause local and systemic inflammation [1,2], resulting in the overactivation of innate immunity. Such overactivation may induce the robust, hyperproduction, and excessive secretion of IFNs, proinflammatory and anti-inflammatory cytokines, and chemokines, leading to cytokine storms. Cytokine storms released during the acute viral infection of distinct viruses such as influenza, coronavirus, Ebola virus, and dengue virus can result in single- or multiple-organ damage and even death [6,7,8,9,10,11,12,13]. Indeed, in COVID-19, the cytokine storm is an important factor leading to the death of many patients [12,13].

The molecular basis of antiviral innate immune signaling is complex, multi-waved, interconnected, and may not always be antiviral. Understanding the complex mechanisms underlying the interactions between viruses and the host innate immune system is key to help develop rational treatment strategies for acute viral infectious diseases, and among them, the JAK-STAT pathway is especially relevant due to its essential role in the regulation of local and systemic inflammation in response to viral infections.

## 2. The JAK-STAT Signaling Pathway in Viral Infections

The JAK-STAT signaling pathway is a transport hub for cytokine secretion and is used by many proinflammatory molecules to mediate the downstream effects and activate gene transcription. The JAK-STAT signaling pathway is mainly composed of three members: tyrosine kinase-related receptors, JAKs, and STATs [14]. Tyrosine kinase-related receptors are transmembrane cytokine receptors that are divided into class I and class II cytokine receptors depending on the specific cytokine families they recognize (the IL-2, IL-3, IL-6, and IL-12/IL-23 cytokine family for type I and IL-10 and the interferon family for type II, respectively) [15]. When cytokines bind to specific receptors, the molecular conformation of JAKs (including JAK1, JAK2, JAK3, and Tyk2) located in the cytoplasm changes, triggering its autophosphorylation or transphosphorylation [16]. Phospho-JAKs result in secondary phosphorylation of the receptors and subsequent docking and phosphorylation of STATs (including STAT1, STAT2, STAT3, STAT4, STAT5a, STAT5b, and STAT6). Phospho-STATs form homodimers or heterodimers that enter the nucleus, bind to DNA, and participate in the regulation of gene transcription. The heterodimer STAT1–STAT2 binds to a third partner, IFN-regulatory factor 9 (IRF9), forming the ISGF3 complex, which, once in the nucleus, binds to specific regulatory sequences, IFN-stimulated response elements (ISRE), to activate the expression of many ISGs. ISGF3 induces most, if not all, of the ISGs encoding effector molecules of cell-intrinsic antiviral defenses such as OAS or MX1. Alternative JAK-STAT pathways include the formation of STAT1 or STAT4 homodimers, which may drive different functional responses to IFN-I [14]. The diversity of these complexes may, in part, explain the broad effects and cell-type specificity of IFN-I-mediated signaling, as it allows the transcription of a wide range of innate and adaptive immune-related genes dedicated to providing protection against viral infection.

**Figure 2:**
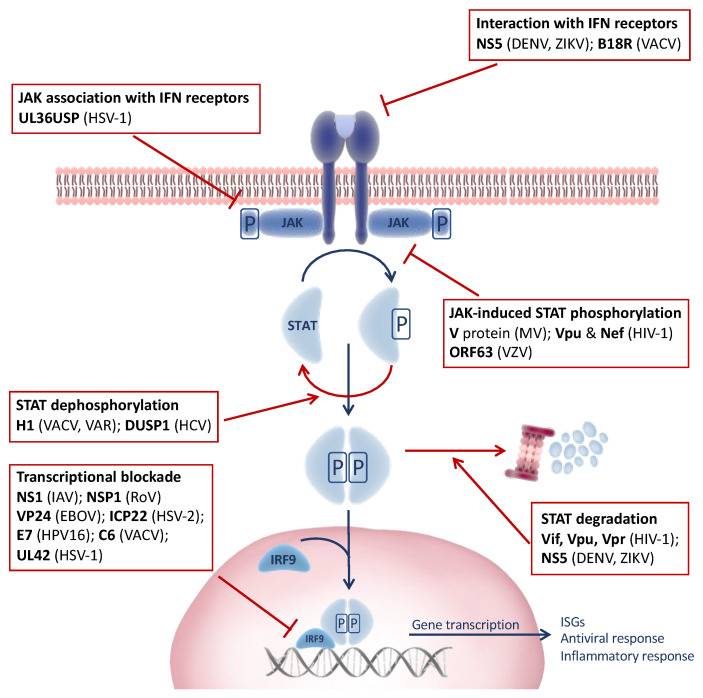
Virus-induced modulation of the Janus kinase–signal transducer and transcriptional activator (JAK-STAT) signaling pathway. Viruses encode several factors that target specific steps in the JAK-STAT signaling pathway through diverse evasion mechanisms to subvert the host immune response. Viral proteins are highlighted in bold and the viruses in parentheses. EBOV, Ebola virus; DENV, dengue virus; HCV, hepatitis C virus; HIV-1, human immunodeficiency virus type 1; HPV16, human papillomavirus type 16; HSV-1, herpes simplex virus type 1; HSV-2, herpes simplex virus type 2; IAV, Influenza A virus; MV, measles virus; RoV, rotavirus; VACV, vaccinia virus; VAR, variola “smallpox” virus; VZV, varicella-zoster virus; ZIKV, Zika virus.

The induction of the JAK-STAT signaling pathway by IFNs leads to the upregulation of hundreds of ISGs, many of which have the ability to rapidly kill viruses within infected cells [4,5]. Since the induction of the antiviral response by IFN is a major threat to virus survival and the JAK-STAT pathway represents a common point governed by a limited group of highly similar proteins, viruses have evolved a myriad of mechanisms to target JAK-STAT signaling in an attempt to counteract host innate immunity (Figure 2).

Several viruses such as dengue virus (DENV) [17,18] and Zika virus (ZIKV) [19] encode proteins targeting different steps of the IFN-JAK-STAT signaling pathway, ranging from the interference of IFN signaling upstream of the JAK-STAT pathway via cytosolic IFN antagonism or the inhibition of intracellular JAK-IFN receptor interactions of the blockade of the JAK-mediated phosphorylation cascade involving the STATs and IRFs. Moreover, some viruses like human immunodeficiency virus type 1 (HIV-1) [20,21] overcome JAK-STAT signaling through proteasomal degradation and dephosphorylation mechanisms. For example, STAT1 and STAT2 are targets for most viruses that manipulate the JAK-STAT pathway in this way. Alternatively, the transcriptional blockade of target gene expression appears to be highly favored by most viruses inhibiting nuclear translocation and formation of the transcription complex ISGF3. In addition, certain viruses might induce the suppressor of cytokine signaling cellular genes (SOCS), a crucial negative regulator of cytokine signaling, preventing the tyrosine phosphorylation of STATs and regulating the pathways in question [14,16].

### 2.1. Viral Interference of IFN Signaling

Many viruses target more than one factor in the IFN signaling pathway, as is the case for paramyxoviruses (reviewed in Reference [22]). Respiratory syncytial virus (RSV) targets both IFN-α/β- and IFN-γ-mediated transcriptional activation by two distinct mechanisms, impairs IFN-β-mediated signaling by inhibiting tyrosine kinase 2 phosphorylation but, also, reduces the nuclear STAT1 interaction with its transcriptional coactivator, resulting in impaired IFN-γ stimulation [23].

In the case of flavivirus, encoding multiple IFN antagonists correlates with high virulence and contributes to their broad host range by overcoming the IFN response in multiple species [24]. The flavivirus genome encodes three structural and seven nonstructural proteins, including the multifunctional factor NS5 that has been identified as a major determinant of virulence and is highly conserved among flaviviruses such as the mosquito-borne flaviviruses DENV and ZIKV. DENV-NS5 blocks the IFN-induced JAK-STAT pathway by interacting with IFN-α/β Receptor Subunit 2 (IFNAR2) and IFN-γ receptors 1 and 2 (IFNGR1 and IFNGR2), interfering with the actions of type 1 and 2 IFNs, which are crucial innate antiviral cytokines (reviewed in Reference [17]).

Similarly, the nonstructural protein 1 (NS1) encoded by the Influenza A virus targets IFN signaling by reducing the transcriptional expression of IFNAR and IFN production [25]. On the other hand, herpes simplex virus type 1 (HSV-1) encodes ubiquitin-specific protease UL36USP, which binds to IFNAR2, dissociating JAK1 from IFNAR2 [26]. Finally, poxviruses such as the Vaccinia virus (VACV) interfere with IFN signaling by encoding IFNAR-like receptor B18R, which acts as a soluble receptor for IFN-α/β, conferring protection against IFN-mediated antiviral activity [27,28].

### 2.2. Blockade of STAT Activation

STAT phosphorylation is a key step in JAK-STAT signaling and the downstream induction of antiviral ISGs. Thus, it is not surprising that viruses commonly develop strategies to antagonize STAT functions [20]. Although, it has become increasingly clear that the modulation of the JAK-STAT pathway is critical for those viruses that establish chronic or persistent infections, many viruses that cause acute infections also target the JAK-STAT pathway [29].

Paramyxoviruses directly interfere with the JAK-STAT signaling pathway through measles virus-encoded V protein (MV-V), which interacts with both JAK1 and STAT1 and blocks the direct phosphorylation of STAT1 by JAK1 [30], similar to HIV-1 accessory proteins Vpu and Nef that blocked STAT1 phosphorylation following IFN-α stimulation, potentially enabling more effective replication in an IFN-α-rich environment [21]. 

Other viruses like the poxvirus VACV instead encode protein phosphatase H1, which reverses STAT1 and STAT2 phosphorylation to block JAK-STAT signaling [31]—likewise, H1-like phosphatase encoded by the highly virulent variola virus (Smallpox) [32]. The deactivation of STATs by dephosphorylation underscores the important role of virus-specific phosphatases in counteracting phosphorylation induced by innate immune signaling pathways that might be crucial for the survival and virulence of these viruses. At least, this is true for the dual-specificity phosphatase 1 (DUSP1) of hepatitis C virus (HCV), which has been demonstrated as being upregulated in the livers of patients with chronic HCV infection refractory to peginterferon (PegIFN) treatment [33]. Silencing DUSP1 in HCV-infected hepatoma cells in vitro enhances the expression of phospho-STAT1 and antiviral ISGs, pointing to a potential treatment strategy against chronic HCV infection [33].

### 2.3. Ubiquitin-Mediated Proteasomal Degradation

Ubiquitination and proteasomal degradation and/or the mislocalization of essential components of the IFN-JAK-STAT pathway is another common strategy shared by several viruses to evade the induction of antiviral ISGs [34,35].

In the case of HIV, the accessory proteins Vif, Vpu, and Vpr are known substrate adaptors for the recruitment of ubiquitin ligase adaptors to cellular target proteins for ubiquitination and proteasomal degradation [36]. In this regard, HIV-1 Vif interferes with effective IFN-α signaling via degradation of the essential constituents of the JAK-STAT pathway, STAT1 and STAT3 [37]. In DENV and ZIKV, NS5 targets STAT2 but not STAT1 for ubiquitination and proteasomal degradation, and this underlines its counteractive measures against IFN-induced antiviral activity [18,19]. Although both DENV-NS5 and ZIKV-NS5 function in a similar manner, ZIKV-NS5 does not require the E3 ubiquitin ligase UBR4 to induce STAT2 degradation, in contrast to DENV-NS5 [19]. Additionally, a recent study has demonstrated the strong induction of the ubiquitin E3 ligase PDLIM2 by HCV, leading to STAT2 degradation [38]. There, the IFN-α-dependent nuclear relocalization of STAT1 and STAT2 resulted in the PDLIM2-mediated ubiquitination and proteasome-dependent degradation of STAT2 but not STAT1 predominantly in the nucleus of HCV-infected cells [38].

### 2.4. Blockade of Transcription Complex Formation

The blockade of ISGF3 complex formation, consisting of the STAT1–STAT2 heterodimer and IRF9 protein, and its transcriptional activity is yet another strategy employed by several viruses to abort active signaling of the IFN response. The porcine reproductive and respiratory syndrome virus (PRRSV) nonstructural protein 1β (nsp1β) blocks the nuclear translocation of STAT1 and STAT2 by inducing the degradation of karyopherin α1 (KPNA1), a critical adaptor in nucleocytoplasmic transport [39]. Similarly, the Ebola virus matrix protein VP24 specifically interacts with KPNA1 but not with the other KPNAs (KPNA2, KPNA3, and KPNA4), resulting in the loss of KPNA1–phospho–STAT interaction and impaired nuclear accumulation of phospho–STAT1 [40]. Moreover, in a recent study, herpes simplex virus type 2 (HSV-2) was shown to antagonize IFN type 1 signaling by encoding a viral protein, ICP22, which blocked ISGF3 nuclear translocation due to the ICP22-induced ubiquitination of STAT1, STAT2, and IRF9 [41].

The targeting of IRF9, a major component of ISGF3, has also been reported for several viruses. Varicella-zoster virus (VZV) ORF63, in addition to inhibiting STAT2 phosphorylation, also targets IRF9 for degradation in a proteasome-dependent manner [42]. Similar to VZV ORF63, Rotavirus NSP1 induces the proteasome-mediated degradation of IRF proteins, including IRF3–IRF9 [43], whereas the human papillomavirus type 16 (HPV16) E7 oncoprotein targets p48, the DNA-binding component of ISGF3, leading to the loss of ISGF3 formation and disruption of IFN-α JAK-STAT signal transduction [44]. Similarly, the UL42 protein of HSV-1 alphaherpesviruses and pseudorabies virus (PRV) suppresses IFN-I signaling by disrupting the association of ISGF3 and ISRE, leading to the decreased transcription and expression of ISGs [45]. Further, downstream of the JAK-STAT signaling pathway, the VACV C6 protein associates with the transactivation domain of STAT2, disrupting the transcriptional complex formation and reducing ISRE-dependent gene expression [46].

### 2.5. Induction of SOCS and Negative Regulation of Cytokine Signaling

Overall, the JAK-STAT pathway is negatively regulated by SOCS proteins through several mechanisms, including the competitive binding of phospho-tyrosine residues with various STAT proteins, inhibition of JAK activity via kinase inhibitory region (KIR) domains, or ubiquitination and subsequent degradation of SOCS-bound elements by the SOCS box [47]. However, some viruses have hijacked this crucial role of SOCS as negative feedback regulators of the JAK-STAT signaling pathway in preventing pathogenesis caused by overstimulation of the immune system to enable them to evade host innate immune responses. For example, the highly contagious and globally endemic HSV-1, characterized by a persistent and lifelong infection, induces the upregulation of SOCS1 and SOCS3 proteins mediated by its viral protein UL13 protein kinase [48]. In fact, the induction of SOCS1 and/or SOCS3 proteins is not limited to HSV-1, as the other human herpesviruses, including VZV, human cytomegalovirus (HCMV), Epstein–Barr virus (EBV), and Kaposi’s sarcoma-associated herpesvirus (KSHV) also induce SOCS1 and/or SOCS3 [33,49,50,51]. Further, modulation of the SOCS protein expression seems to be an important mechanism for the persistence of chronic HCV infection, as it encodes several proteins targeting SOCS. HCV protein p7 induces SOCS3 via STAT3 and extracellular signal-regulated kinase (ERK) activation, leading to the suppression of the TNF-α-specific inflammatory response [52]. In addition, the HCV core protein has differing effects on SOCS1 and SOCS3: induces SOCS3-mediated impairment of IFN-α-induced signal transduction and inhibits STAT1 phosphorylation [53] but exerts inhibitory effects on SOCS1 expression [54].

In summary, the extraordinary diversity of strategies that viruses have evolved to interfere with JAK-STAT signaling stress the relevance of this pathway as a putative antiviral target for the design of new antiviral drugs, alternative therapeutic strategies, or as adjuvants for live attenuated vaccines.

## 3. Therapeutic Strategies Targeting JAK-STAT Signaling Pathway as Modulators of Viral Infections

Although activation of the JAK-STAT pathway primarily promotes the upregulation of immune-related genes against infections and cancer, dysregulated immunity and interferonopathies could lead to several immune disorders, ranging from autoimmunity or allergic diseases to autoinflammatory diseases and cancer [15]. Soon after their discovery, JAKs were quickly identified as therapeutic targets. The first JAK inhibitors (JAKi) were approved about a decade ago, and now, there are eight JAKi approved for the treatment of rheumatologic, dermatologic, hematologic, and gastrointestinal indications, along with an emergency authorization for COVID-19, the latter being the first JAKi used for the treatment of a viral infection [55].

JAKi are generally safe and effective. The group of inhibitory molecules were initially developed to exert their effects by blocking the ATP-binding pocket of the JAK catalytic domain. Although these compounds are relatively selective, with limited off-target effects compared to other kinases, these JAKi block the activity of multiple JAKs both in vitro and in vivo (reviewed in Reference [56]). Thus, several more selective JAKi, along with agents that target kinase families beyond JAKs, are being developed, opening the door to more specific treatments that might significantly impact the treatment of viral infections.

As described above, JAK-STAT modulation by a plethora of distinct viruses underlines the importance of this pathway as a putative antiviral strategy. In this regard, multiple associations between JAK inhibitors and viruses have been described (Table 1).

Recently, the FDA-approved compounds ruxolitinib, tofacitinib, baricitinib, and filgocitinib have been proposed as antiviral agents against human immunodeficiency virus (HIV) independently of their original clinical indications [57,58,59]. Using high-throughput drug screening, ruxolitinib and filgotinib were identified as putative inhibitors of HIV-1 transcription through the blockade of splicing, reducing the proliferation of HIV-1-infected cells and blocking HIV-1 latency reactivation through the suppression of T-cell activation pathways [60]. Similar effects on T-cell activation were proposed for ruxolitinib and baricitinib in a HIV latency model [57], along with the induction of proapoptotic protein BCL-2, therefore suggesting the ability of these JAKi to block HIV reactivation and reduce the latent reservoir. Moreover, other authors have also claimed that ruxolitinib and tofacitinib exert their anti-HIV activity by inhibiting both HIV-1 replication and HIV-1 latency reversal [58,59]. The suggested mechanisms for these observations include the inhibition of IL-6 and TNFα production, which, in turn, blocks viral gene transcription and HIV-1 replication, the blockade of STAT phosphorylation and the subsequent binding to HIV LTR, inhibiting viral gene transcription and the inhibition of T-cell activation and proliferation.

Antiviral activity against other viruses has also been described for other JAKi. The phase II compound AT9283 was found to decrease HSV-1 infection in neurons by 75–95% [61], although no mechanism was proposed. Another JAKi in the phase II clinical stage, cerdulatinib, was also evaluated in cells infected and transformed with the HTLV-1 virus, which causes adult T-cell leukemia/lymphoma [62], resulting in cell cycle arrest in G2/M and the induction of cell apoptosis, preferentially in infected cells by suppressing the AKT serine–threonine protein kinase, ERK, activator protein 1 (AP-1), STAT, and NF-κB pathways.

**Table 1:** Reported effects of JAKi on viral infections.

JAK Inhibitor		Target	Modulation of Viral Infection		Indication/s *	Ref
			Negative/ Antiviral	Positive/ Proviral		
Approved	Ruxolitinib	JAK2/1	HIV, SARS-CoV-2	VZV, HCMV, HBV, EBV, HHV-6, BKV, HSV, HPyV2	Myelofibrosis Polycythaemia vera Graft-versus-host disease Atopic dermatitis	[57,58,59,63,64,65,66,67,68,69,70]
	Tofacitinib	JAK3/2	HIV, SARS-CoV-2	VZV, HCMV, BKV, HBV	Rheumatoid arthritis Ulcerative colitis	[58,71,72,73,74,75]
	Baricitinib	JAK1/2/ Tyk2	HIV, SARS-CoV-2	VZV, HCMV, HBV, EBV, HEV	Rheumatoid arthritis Atopic dermatitis COVID-19 ^#^	[55,68,75,76,77,78,79,80,81,82]
	Fedratinib	JAK2/FLT3/RET/BRD4	SARS-CoV-2	-	Myelofibrosis	[68,83,84]
	Filgotinib	JAK1/2/ Tyk2	HIV	VZV	Rheumatoid arthritis	[60,75]
	Upadacitinib	JAK1/2	-	VZV	Rheumatoid and psoriatic arthritis Ankylosing spondylitis Atopic dermatitis	[74]
	Peficitinib	panJAK	-	VZV	Rheumatoid arthritis	[74]
	Delgocitinib	panJAK	-	-	Atopic dermatitis	
Phase III	Pacritinib	JAK2/FLT3/Tyk2	SARS-CoV-2	-	Myelofibrosis	[82,85]
	Lestaurtinib	JAK2/FLT3/TrkA	SARS-CoV-2	-	Acute lymphoblastic leukemia	[86]
	Decernotinib	JAK3	-	VZV	Rheumatoid arthritis	[74]
	Jaktinib	JAK1/2	SARS-CoV-2	-	Myelofibrosis Alopecia areata	[87]
Phase II	Cerdulatinib	panJAK/Syk	HTLV-1	-	Vitiligo Chronic lymphocytic leukemia Non-Hodgkin’s lymphoma	[62]
	Nezulcitinib	panJAK	SARS-CoV-2	-	Acute lung injury COVID19	[88]
	AT9283	JAK2/3/2/ Aurora A/B	HSV-1	-	Multiple myeloma	[61]

HIV, human immunodeficiency virus; SARS-CoV-2, severe acute respiratory syndrome coronavirus 2; HTLV-1, human T-lymphotropic virus type 1; HSV-1, herpes simplex virus 1; VZV, varicella zoster virus; HCMV, human cytomegalovirus; HBV, hepatitis B virus; EBV, Epstein–Barr virus; HHV-6, human herpesvirus 6; BKV, BK virus/polyomavirus hominis 1; HSV, herpes simplex virus; HPyV2, human polyomavirus 2; HEV, hepatitis E virus. ^*^ According to FDA authorization or the latest ongoing clinical trials. # Emergency use authorization.

A current concern regarding viral infections is the control of SARS-CoV-2, the coronavirus responsible for the COVID-19 pandemic. In the most severe cases, SARS-CoV-2 infection prompts a cytokine storm that is crucial for the development of acute respiratory distress syndrome (ARDS) that may, ultimately, lead to multiple organ failure and even death [89]. Given the implication of the JAK-STAT pathway in the production of proinflammatory cytokines, the repurposing of several JAK inhibitors has been proposed to ameliorate COVID-19 symptomatology (reviewed elsewhere in References [90,91,92]). Among them, baricitinib was authorized for emergency use to treat hospitalized COVID-19 patients due to its capacity to block the JAK-STAT signaling pathway and the subsequent overproduction of cytokines in severe patients but also affecting viral endocytosis [79]. The infection of lung cells by SARS-CoV-2 is mediated by the binding to the cellular angiotensin-converting enzyme II (ACE2) receptor and the priming of the viral S protein by transmembrane serine protease 2 (TMPRSS2) [93,94]. ACE2 has several regulators that mediate endocytosis, two of them being AP2-associated protein kinase-1 (AAK1) and cyclin G-associated kinase (GAK). The JAK inhibitor baricitinib has been shown to bind and interact with both AAK1 and GAK [95] and to block viral entry [79], suggesting a novel antiviral mechanism for baricitinib as an entry inhibitor against SARS-CoV-2 infection [77]. A similar effect has been proposed for ruxolitinib and fedratinib [68]. In addition to these findings, JAK inhibitor pacritinib has also been proposed as a putative inhibitor of ACE2 and TMPRSS2 in an in silico study [82]. The hypothetical role of JAK inhibitors as both modulators of the cytokine storm and inhibitors of SARS-CoV-2 entry has prompted their study in clinical trials with COVID-19 patients. In fact, clinical trials have already started for JAK inhibitors tofacitinib (NCT04469114) [71], nezulcitinib (NCT04402866) [88], jaktinib (ChiCTR2000030170) [87], and baricitinib (NCT04421027). Clinical trials for JAK inhibitors ruxolitinib (NCT04362137) and pacritinib (NCT04404361) have also been reported; however, both have been terminated, as no clinical benefit was observed in comparison to the standard of care [85,96]. 

In contrast to the proposed antiviral role, treatment with JAK inhibitors for immune-mediated inflammatory diseases, such as rheumatoid arthritis, has also been associated with an increased risk of infections, including those of viral origin, due to their immunosuppressive effects [63,65,66,67,69,70,72,73,74,76,78]. In this regard, the incidence of herpes zoster infection caused by the reactivation of the latent varicella-zoster virus (VZV) has been reported in several clinical trials and reviewed elsewhere [74]. VZV reactivation cases have been documented in the clinical trials of several JAKi, including tofacitinib, baricitinib, upadacitinib, filgotinib, decernotinib, and peficitinib, although incidences vary depending on the compound and the study population. Another case of VZV reactivation, this time associated with meningoencephalitis, was also reported in a myelofibrosis patient treated with ruxolitinib [70]. Other viral infections reported in clinical trials include HCMV during baricitinib and tofacitinib treatment [73,76] and also in a case report from a myelofibrosis patient receiving ruxolitinib [70]. Reactivation of latent hepatitis B virus (HBV) has been observed in immunosuppressive treatments, and therefore, several studies have evaluated the incidence of this event in clinical trials and/or patients using JAK inhibitors. As expected, a positive correlation for HBV reactivation and JAK inhibitor treatment was found in baricitinib [78] and tofacitinib [72] and, also, in a prospective study with myeloproliferative neoplasm patients treated with ruxolitinib [69]. Human BK polyomavirus (BKV) is a virus that rarely causes disease, except in immunocompromised or immunosuppressed individuals. In the context of JAK inhibitors, one case of BKV encephalopathy was reported in a tofacitinib clinical trial with rheumatoid arthritis patients [72], and two more cases presented detectable levels of BKV viruria in a study with ruxolitinib-treated patients for acute corticosteroid-refractory graft-versus-host disease (SR-GVHD) after hematopoietic cell transplantation [65]. Although not common, infection with the opportunistic pathogen EBV, EBV reactivation, and EBV-driven suspected lymphoproliferative disorder have also been described, being associated with baricitinib [77] and ruxolitinib treatment [66], respectively. Other infections reported in ruxolitinib regimens include two cases of human herpesvirus 6 (HHV-6) viremia in acute SR-GVHD patients and HSV-1 with oral ulcerations in a chronic SR-GVHD patient [65], HSV reactivation and disseminated infection in a patient with myelodysplastic syndrome [63], and an association between meningitis and human polyomavirus 2 (HPyV2/JC virus) reactivation fourteen days after treatment initiation [67]. For the FDA-approved compound baricitinib, infection with hepatitis E virus (HEV) was also reported in a RA patient under treatment [78].

Overall, the fact that JAKi can provide antiviral effects at the approved therapeutic dose is an undeniable advantage over other potential inhibitors of viral infections targeting host cellular pathways. However, some concerns could arise from the best-known aspects of the mechanisms of action of these drugs, mainly derived from the impairment of interferon-mediated antiviral responses, potentially facilitating the susceptibility and evolution of certain uncommon and chronic viral infections.

## 4. Conclusions

The rapid emergence and dissemination of SARS-CoV-2 and subsequent COVID-19 pandemic has placed an excessive burden on worldwide healthcare systems and has also led to dramatic consequences for the global economy. Thus, therapeutic approaches aimed at mitigating viral outbreaks are of utmost global priority, both to improve clinical outcomes for the current globally devastating virus but also to expand the number of effective antiviral treatments to fight future viral outbreaks. In this sense, JAKi represent a potential novel antiviral treatment for viral infections, due to their proven efficacy against diseases with excessive cytokine release and their direct antiviral activity against distinct viruses, including coronaviruses.

As described above, JAKi are powerful therapeutics, and during the past decade, remarkable progress has been achieved in the use of JAKi for numerous disorders. While it was reasonably predictable that JAKi would be efficacious in treating certain inflammatory diseases, their proven efficacy and safety in a broad spectrum of disorders is remarkable, comparable to biologics with much more focused targets, therefore representing good candidates for drug-repurposing strategies, including viral infections.

Innate immunity provides common first-line protection against invading pathogens. When a virus infects a human, their innate immunity is the first to be activated, consequently promoting the activation of the adaptive immunity to prevent the development of the disease. However, in some cases, this is not achieved, and the infection thrives as the disease develops or, on the contrary, results in the excessive uncontrolled release of proinflammatory cytokines that can lead to life-threatening systemic inflammatory syndromes involving the elevated levels of circulating cytokines and immune cell hyperactivation. Several viruses, such as SARS-CoV, MERS-CoV, avian influenza, and the Ebola virus, are known to hyper-stimulate the immune system, inducing a cytokine storm, in which the elevated levels of cytokines and chemokines have been linked to disease severity and clinical progression [6,7,8,9,10,11,12,13]. Thus, it would seem logical to target this response in order to reduce the self-inflicted damage initiated by the host in response to infection. Yet, to date, successfully targeting the immune system during acute infection has proven extraordinarily difficult and largely unsuccessful. JAK inhibitors play a vital role in this regard, as they are highly effective at reducing type I IFN-driven inflammation, and the current preclinical and clinical data demonstrate their efficacy as a COVID-19 treatment, paving the way towards their use as treatment strategies against other viral infections.

However, there are still many unanswered questions on the feasibility of using JAK inhibitors as antivirals, including the number and characteristics of viral infections that might benefit from targeting the JAK-STAT signaling pathway. Moreover, treatments with JAK inhibitors have also been linked to immune suppression and complications associated with viral infections, demonstrating again the critical equilibrium of an effective host immune response and the devastating effect of immune dysregulation. Thus, additional preclinical and clinical research is needed to carefully determine the specific antiviral mechanism for each pathogen, the correct dose for the distinct indications, the best timing of administration, and their putative efficacy against acute and chronic viral diseases.

Infectious diseases still pose a significant threat globally, accounting for approximately half of all deaths in the world. As economies develop, urbanization and environmental degradation gather pace, and the structures of societies change with many new challenges, hindering the fight against the emergence of new diseases but, also, the continued rise of drug resistance by both viral and bacterial infections that are outpacing the rate of discovery of new treatments. Against this backdrop of drug resistance and the emergence of new pathogens, increasing interest has focused on the development of drugs that target the immune responses to infections, and JAK inhibitors will most likely play a relevant role in the nearest future in this regard. More JAK inhibitors with improved selectivity and limited risk for the existing latent infections are being developed and will certainly come along. We envisage that patients with certain viral infections where type I IFN-driven inflammation and pathology contribute to severity might benefit from the suppression of unwanted type I IFN-driven inflammation—most importantly, alleviating this immunopathology without affecting the antiviral effects of innate immune activation.

## Data Availability

No new data were created or analyzed in this study. Data sharing is not applicable to this article.
